# Novel 1,2,3-Triazole-Based Benzothiazole Derivatives: Efficient Synthesis, DFT, Molecular Docking, and ADMET Studies

**DOI:** 10.3390/molecules27238555

**Published:** 2022-12-05

**Authors:** Zohreh Mirjafary, Mahdieh Mohammad Karbasi, Parsa Hesamzadeh, Hamid Reza Shaker, Asghar Amiri, Hamid Saeidian

**Affiliations:** 1 Department of Chemistry, Science and Research Branch, Islamic Azad University, Tehran 14515-775, Iran; 2 Department of Science, Payame Noor University (PNU), Tehran P.O. Box 19395-4697, Iran

**Keywords:** 1,2,3-triazole, click reaction, cycloaddition, DFT calculation, molecular docking, ADMET study

## Abstract

A new series of 1,2,3-triazole derivatives **5a**–**f** based on benzothiazole were synthesized by the 1,3-dipolar cycloaddition reaction of S-propargyl mercaptobenzothiazole and α-halo ester/amide in moderate to good yields (47–75%). The structure of all products was characterized by ^1^H NMR, ^13^C NMR, and CHN elemental data. This protocol is easy and green and proceeds under mild and green reaction conditions with available starting materials. The structural and electronic analysis and ^1^H and ^13^C chemical shifts of the characterized structure of **5e** were also calculated by applying the B3LYP/6-31 + G(d, *p*) level of density functional theory (DFT) method. In the final section, all the synthesized compounds were evaluated for their anti-inflammatory activity by biochemical COX-2 inhibition, antifungal inhibition with CYP51, anti-tuberculosis target protein ENR, DPRE1, pks13, and Thymidylate kinase by molecular docking studies. The ADMET analysis of the molecules **5a**–**f** revealed that **5d** and **5a** are the most-promising drug-like molecules out of the six synthesized molecules.

## 1. Introduction

Among the types of heterocycles, systems containing nitrogen, oxygen, and sulfur atoms have the most activity and biological aspects [1]. So far, many of these organic compounds that have pharmacological activity have been synthesized and used [2]. Five-membered rings with two carbon and three nitrogen atoms are called triazoles. Triazoles have two types of isomers: 1,2,3-triazole and 1,2,4-triazole. Using a 1,3-dipolar cycloaddition reaction between alkynes and aryl/alkyl azides, 1,2,3-triazoles can be easily synthesized as two isomers of 1,4- and 1,5-disubstituted triazoles [3,4,5,6]. In 2002, Meldal and Sharples showed that the 1,3-dipolar cycloaddition reaction between an azide and terminal alkyne in the presence of copper (I) resulted in the regioselective synthesis of 1,4 disubstituted-1,2,3-triazoles [7,8]. 1,2,3-Triazoles have been used to prepare commercial and clinical drugs [9,10]. Some of the biological properties of these compounds are anti-epileptic, anti-cancer, anti-viral, anti-microbial, anti-fungal, anti-tumor, anti-diabetic, anti-tuberculosis, anti-inflammatory, anti-HIV, and anti-allergic [10,11,12,13,14,15,16]. The application of benzotriazole derivatives as polymer field effect transistor materials was also reported [17]. Benzothiazoles are also vital heterocyclic compounds found in many natural products. Several clinical benzothiazole-based drugs have been found to be effective in treating various diseases. These compounds have medicinal and biological properties, including anti-cancer, anti-tuberculosis, anti-HIV, anti-fungal, anti-bacterial, anti-diabetic, anti-malarial, anti-inflammatory, antioxidant, and antihistamine [18,19,20,21,22]. Benzothiazoles have many applications in polymers and dyes [23,24,25]. 2-Mercaptobenzothiazole acts as an accelerator in rubber vulcanization [26].

**Scheme 1:**
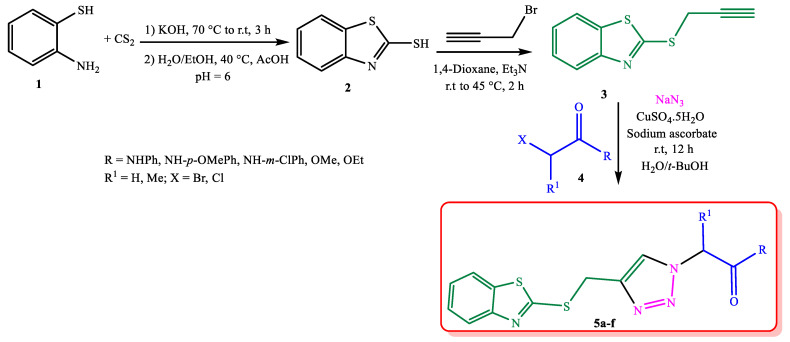
Efficient synthesis of novel 1,2,3-triazoles bearing the benzothiazole group **5a**–**f**.

Due to the importance and application of 1,2,3-triazoles bearing the benzothiazole group [27,28], the synthesis of these derivatives is essential for organic chemists and biologists, and efforts are underway to design drugs based on these compounds with high activity and lower toxicity. As a result of our group’s experience synthesizing 1,4-disubstituted 1,2,3-triazole derivatives [29,30,31,32,33,34], the new 1,2,3-triazoles were prepared using benzothiazole groups **5a**–**f** via a 1,3-dipolar cycloaddition of *S*-propargylated mercaptobenzothiazole **3**, sodium azide, and α-halo ester/amide **4** in the presence of CuSO_4_/sodium ascorbate in a mixture of H_2_O/*t*-BuOH (Scheme 1). The density functional theory (DFT) calculations, with the B3LYP/6-31 + G(d, *p*) method, were applied to investigate the physicochemical properties of the characterized structure of **5e**, including structural, electronic, and spectral analysis. Moreover, the study concludes by examining each of the synthesized compounds to specific targets using molecular docking to predict the possible biological activity of the proposed molecules. As mentioned above, triazoles have anti-inflammatory, anti-tuberculosis, and anti-fungal properties. For this purpose, the COX-2 protein was chosen for the anti-inflammatory target. COX-2 has been an important target for pain reduction drugs through the years and even shows some evidence relating this protein to cancer [35,36,37]. Numerous studies have been conducted on this protein with triazole structures in silico, in vitro, and in vivo that have shown promising results [38,39]. For anti-tuberculosis activity, four different targets were selected. DPRE1 has been one of the potential targets for resistant tuberculosis. Anti-tuberculosis target protein ENR was another protein target on our list. The thymidylate kinase of Mycobacterium tuberculosis and Pks13 are the other anti-tuberculosis targets we selected for this research due to their critical role in this disease. For anti-fungal activity, we analyzed our synthesized molecule’s activity against Saccharomyces cerevisiae CYP51 [40,41,42,43,44,45,46,47,48,49,50,51]. Absorption, distribution, metabolism, excretion, and toxicities (ADMET) prediction is one of the essential steps for drug discovery and development. After the docking study, we analyzed the molecules **5a**–**f** for ADMET properties to analyze and predict these molecules against the control molecules for each target to better understand the chances of introducing them to the next phase of drug discovery and development. Our molecular docking experiments for the molecule **5e**, which has a chiral center, were conducted to check how the enantiomers affect its potential activity and interactions with proteins. Due to the limitations of ADMET servers, which predict properties based on the 2D structure of molecules, we could not generate separate properties for each of the two enantiomers.

## 2. Results and Discussion

### 2.1. Synthesis and Spectroscopic Characterization of 1,2,3-Triazoles ***5a***–***f***

2-Mercaptobenzothiazole **2** was obtained according to the literature [52] and then was propargylated by propargyl bromide at 45 °C in dioxane in the presence of Et_3_N (Scheme 1). The azide intermediates were obtained by the *in situ* reaction of the corresponding α-halo ester/amide **4** with sodium azide. The desired 1,2,3-triazoles **5a**–**f** were prepared by the azide-alkyne [3 + 2] cycloaddition reaction at room temperature. To optimize the reaction conditions, the solvent, catalyst, and temperature effect were investigated on the model reaction of **3** with **4a** in the presence of sodium azide (Table 1). It should be mentioned that increasing the temperature had no good effect on the yield of the reaction (Entries 1–3 and 5, Table 1). With the inclusion of a low amount of copper catalyst (30 or 10 mol%), the reaction took longer, as well as resulted in a low yield of the product. CuSO_4_ (50 mol%) as a catalyst, sodium ascorbate as a reductant agent (50 mol%), and H_2_O/*t*-BuOH (1:1) as solvent at room temperature were applied as the best reaction conditions (Entry 4, Table 1).

**Scheme 2:**
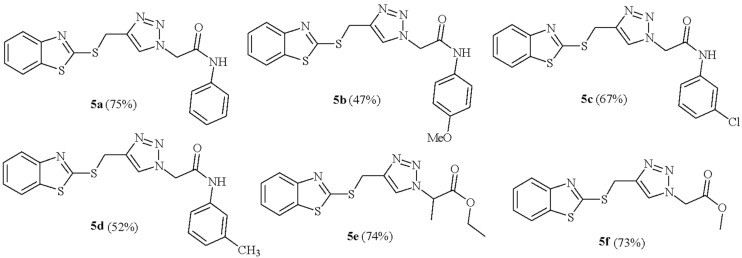
The structure of the synthesized 1,2,3-triazole derivatives **5a**–**f**.

The generality of the protocol was then studied. As shown in Scheme 2, 1,2,3-triazole derivatives (**5a**–**f**) were synthesized in moderate to good yields (47–75%) by the reaction of a series of α-halo ester/amide **4** and *S*-propargyl mercaptobenzothiazole **3**. The structure of triazoles **5a**–**f** was established based on ^1^H NMR, ^13^C NMR spectral data, and CHN analysis. As a representative example, the ^1^H NMR and ^13^C NMR data of the product **5b** are discussed. The ^1^H NMR spectrum of **5b** consisted of a singlet peak at δ = 3.72 ppm for the hydrogens of the methoxy group, a broad line at δ = 4.75 ppm for the methylene of the -CH_2_S- group, a broad line at δ = 5.27 ppm for –CH_2_CO-, eight aromatic hydrogens at δ = 6.91–8.04 ppm, a sharp characteristic signal at δ = 8.16 ppm for hydrogen on the triazole ring, and a broad line at δ = 10.34 ppm correlating with –CONH (amidic hydrogen). The amidic hydrogen (acceptor) (N-H…N) forms a strong intramolecular hydrogen bond with the nitrogen atom of the triazole ring (donor), resulting in a deshielded proton for the amide moiety in ^1^H NMR. The ^1^H-decoupled ^13^ CNMR spectrum of **5b** showed 17 distinct resonances, a resonance at δ = 166.30 ppm for the carbon of carbonyl group, 13 distinct resonances for aromatic carbons between δ = 114.44–164.02 ppm, two peaks at δ = 55.60 and 52.61 ppm for the carbons of the methylene groups, and a resonance at δ = 27.85 ppm for the carbon of the methoxy group, which is in agreement with the proposed structure.

### 2.2. Theoretical Studies on the 1,2,3-Triazole ***5e***

In continuation, the physicochemical properties of **5e**, such as structural, electronic, and spectral data, were investigated by the DFT-B3LYP computational method in conjunction with the 6–31 + G(d, *p*) basis set.

#### 2.2.1. Benchmarking of the Computational Method

DFT calculations have successfully provided the theoretical background of popular qualitative chemical concepts [53]. Furthermore, organic chemists use the DFT-B3LYP method for various chemical calculations [54,55,56,57]. This method has been utilized in the present study for performing calculations. The experimental ^1^H NMR and ^13^C NMR data for **5e** could pave the way to detect the accurate computational method.

The experimental ^1^H NMR spectrum of **5e** consisted of a triplet at δ = 1.10 for methyl, a doublet for methyl at 1.72, a multiplet at 4.41 for methylene (-OCH_2_-), and a singlet at 4.71 for methylene (-SCH_2_−), a quartet at 5.62 for –NCH- (1H), four protons for phenyl rings at δ = 7.35–7.99 ppm, and a characteristic signal at δ = 8.24 ppm for hydrogen on the triazole ring. The ^1^H-decoupled ^13^C NMR spectrum of **5e** showed 15 resonances, which is in agreement with the proposed structure, while the carbonyl appearing at δ = 169.21 ppm, nine distinct resonances for the aromatic carbons of phenyl and triazole rings between δ = 121.30–165.74 ppm, and five resonances at δ = 13.80–61.66 ppm for aliphatic carbons. The simulated NMR spectra for **5e** were obtained at the B3LYP/6-31 + G(d, *p*) level with the aid of the Gauss View software [58]. Table 2 summarizes the calculated and experimental ^1^H and ^13^C chemical shifts for **5e**. The ^1^H and ^13^C chemical shifts are reported in parts per million (ppm) relative to tetramethylsilane as the reference, and its corresponding shifts were calculated at the same theoretical level. For the 6-31 + G(d, *p*)-optimized geometry of **5e** in DMSO, the NMR chemical shifts were also calculated using the same basis set and solvent as the experimental NMR spectra.

**Table 2:** B3LYP/6-31 + G(d, *p*) calculated and experimental ^1^H and ^13^C chemical shifts (in ppm) of **5e** in DMSO.

Atom ^a^	δ_B3LYP_	δ_exp_	Atom ^a^	δ_B3LYP_	δ_exp_	Atom ^a^	δ_B3LYP_	δ_exp_
H^35−37^	1.27	1.01	H^19^	8.15	8.01	C^4^	119.53	121.82
H^27−29^	1.83	1.72	H^7^	8.17	8.24	C^6^	120.86	123.85
H^32^	4.10	4.10	C^34^	16.54	13.80	C^18^	123.18	124.59
H^16^	4.55	4.71	C^26^	20.67	17.05	C^5^	123.45	126.41
H^25^	5.55	5.62	C^15^	35.50	27.44	C^2^	139.12	134.76
H^10^	7.62	7.35	C^24^	64.14	57.53	C^20^	140.05	142.29
H^9^	7.77	7.49	C^31^	66.31	61.66			
H^8^	8.09	7.90	C^1^	118.96	120.31			

^a^ For the numbering of atoms, refer to Figure 1.

**Figure 1:**
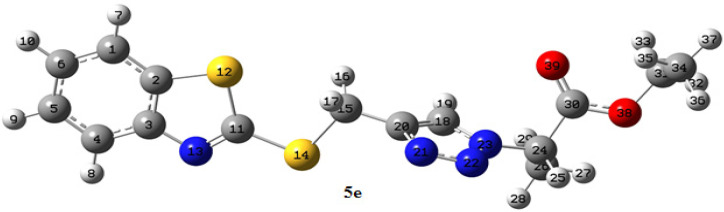
B3LYP/6-31 + G(d, *p*) optimized geometry of **5e** in DMSO.

**Figure 2:**
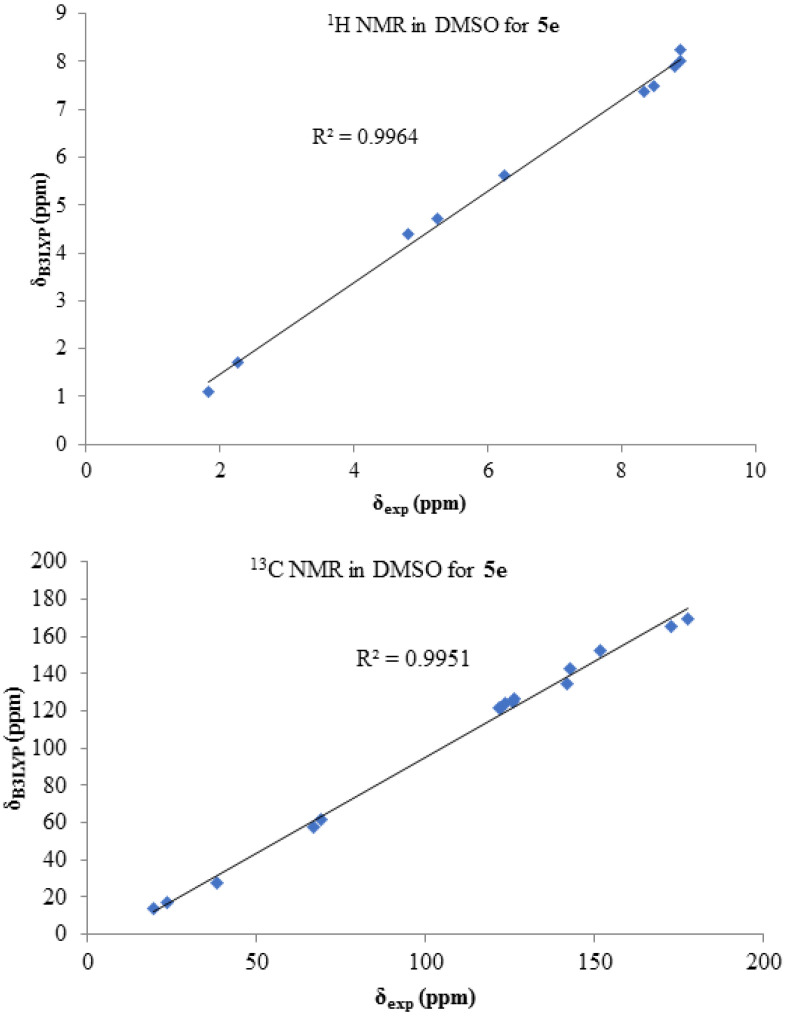
^1^H and ^13^C chemical shifts’ correlation between calculated and experimental data for **5e** in DMSO.

The calculated ^1^H and ^13^C chemical shifts are in excellent agreement with the experimental data; regression was obtained R^2^ = 0.996 for ^1^H NMR and R^2^ = 0.995 for ^13^C NMR data (Figure 2). ^1^H NMR comparison showed a better correlation rather than ^13^C NMR. Based on the results, it is possible to continue the calculations and obtain the physicochemical properties of **5e** using the B3LYP/6–31 + G(d, *p*) method.

#### 2.2.2. Structural Characterization of **5a** and **5e**

Figure 1 illustrates the optimized geometry of **5e** at the B3LYP/6-31 + G(d, *p*) computational level in DMSO. Table 3 shows the representative calculated bond lengths and bond angles for **5e**, which are within normal ranges. The bond C = O was 1.215 Å. The single S-C bonds extend in the range of 1.757–1.856 Å. The C^20^-C^18^-N^23^ and C^1^-C^2^-N^13^ angles are 104.6 and 115.3°. In the esteric group, C^24^-C^30^-O^38^ was calculated as 109.7°, while the O^39^-C^30^-O^38^ resulted as 125.5°.

**Figure 3:**
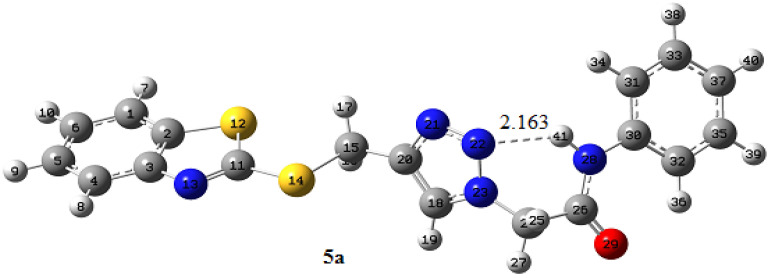
The B3LYP/6-31 + G(d, *p*) optimized geometry of **5a** (distance in Å).

As mentioned above, there is strong intramolecular hydrogen bonding (IMHB) between the amidic hydrogen and nitrogen atom of the triazole ring (N-H…N) in the chemical structures of **5a**–**d**. This interaction was confirmed from the ^1^H NMR spectra of **5a**–**d**, resulting in a deshielded proton for the amide group. As can be seen from the optimized structures of **5a** (Figure 3), there is a very strong IMHB with the hydrogen atom of the amide group as the acceptor center and the nitrogen atom of the triazole ring as the donor site. By analyzing the natural bond orbital (NBO) of **5a**, we can gain valuable insight into the intermolecular/intramolecular interactions, such as hydrogen bonding, numerically according to two-order perturbation energy (E^(2)^) [59]. The LP (1) N^22^ → BD*(1) N^28^-H^41^ as a remarkable interaction with energy of 4.85 kcal mol^−1^ in **5a** is attributed to the IMHB interaction. The structures of **5b**–**d** exhibit also an analogous trend.

#### 2.2.3. Electronic Characterization of **5e**

Table 4 shows the electronic data calculated by the B3LYP/6-31 + G(d, *p*) method of **5e** in DMSO. The dipole moment in DMSO for **5e** is 5.81 Debye, indicating the high polarity. The presence of polar groups, such as the amide group and unsymmetrical structure, are the reasons for this high dipolar moment data.

**Figure 4:**
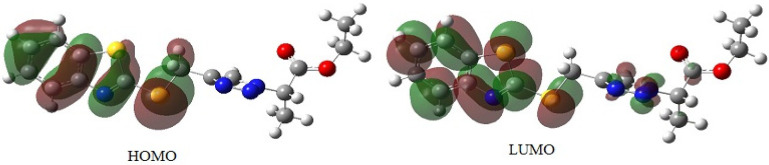
HOMO and LUMO for **5e** calculated by B3LYP/6-31 + G(d, *p*) level.

The highest occupied molecular orbital (HOMO) and lowest unoccupied molecular orbital (LUMO) are the frontier orbitals that reveal the ability to donate or accept an electron, respectively. It is worth mentioning that removing electrons from HOMO is easier, and this orbital can donate electrons. As shown in Figure 4, the HOMO and LUMO orbitals are localized mainly on the benzothiazole ring. A useful parameter for determining the organic compound’s reactivity is the gap between the HOMO and LUMO energy levels (E_g_). The difference between E_HOMO_ and E_LUMO_ (E_g_) is 4.98 eV (Table 4).

The global electrophilicity index (GEI) is a criterion of the molecule’s ability to accept electrons, which has been used to determine some adequate information on the structure, reactivity, dynamics, aromaticity, Lewis acidity, and toxicity [60,61]. Parr defined the GEI (ω) as [62]:

ω = μ^2^/2η (1)

**Figure 5:**
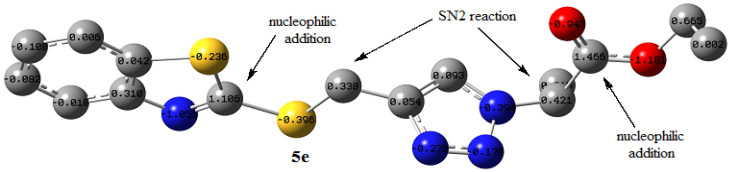
Possible nucleophilic sites in **5e**, based on NBO analysis and the electrophilicity index (hydrogen atoms are omitted for clarity).

μ = (E_LUMO_ + E_HOMO_)/2 and η = (E_LUMO_ − E_HOMO_)/2 (2)

where μ and η are the chemical potential and hardness, respectively, given by Equation (2); **5e** has a GEI of 2.94 eV. The GEI can be categorized based on Domingo’s scale [63]. Electrophiles are divided into three groups based on the GEI value: strong, mild, and weak. When the electrophilic index ω is more than 1.5 eV, the electrophile is strong; in the range of 0.8 to 1.5 eV, it is mild, and when it is less than 0.8 eV, it is a weak electrophile. Based on Domingo’s scale, the compound **5e** is a strong electrophile and can easily interact with a typical nucleophile. The NBO charge distribution for the structure of **5e** is shown in Figure 5. According to the NBO analysis, there is a remarkable excess of positive charge (1.466 e) on the carbon atom of the carbonyl group. The study also indicates that the carbon atom of the imine unit (N = C) in the benzothiazole ring, methylene (-SCH_2_-), and –NCH- bear a positive charge of 1.106, 0.338, and 0.421e, respectively. NBO analysis and the electrophilicity index of **5e** show that a nucleophile can attack these positive carbon centers. Possible nucleophilic centers in **5e**, based on NBO analysis and the electrophilicity index, are shown in Figure 5.

### 2.3. Molecular Docking and ADMET Studies

Molecular docking results show that molecule **5d** has the highest binding energy for 1G3U (−9.7 kcal mol^−1^), 1QSG (−9.1 kcal mol^−1^), and 5F19 (−9.6 kcal mol^−1^), even more than the control molecules. Based on this information, **5d** is likely to be a promising inhibitor of the proposed target. Molecule **5b** has the same binding energy as **5d** for 1QSG, giving the idea of the potential anti-tuberculosis activity of this molecule. In 4ZE2, the binding energy of the control molecule is similar to **5a**, equal to −9.7 kcal mol^−1^. The synthesized molecules **5a**–**f** show higher binding energy and more minor interactions with two other proteins (4P8N, 5V3X), indicating that these molecules have a lower anti-tuberculosis activity than their reference molecules. In 4P8N, the control molecule BTZ043 shows a binding energy of −10.6 kcal mol^−1^. In addition, **5d** has the lowest binding energy of all proposed molecules, with a binding energy of −9.6 kcal mol^−1^. As shown by the binding energy of 5V3X, the reference molecule has a binding energy of −10.1 kcal mol^−1^, which indicates a strong interaction between this molecule and the protein target. Given that the six synthesized molecules do not bypass the reference molecule and have lower inhibitory activity against the target, **5d** has the best binding energy of −9.7 kcal mol^−1^. A summary of the docking results can be found in Table 5.

**Figure 6:**
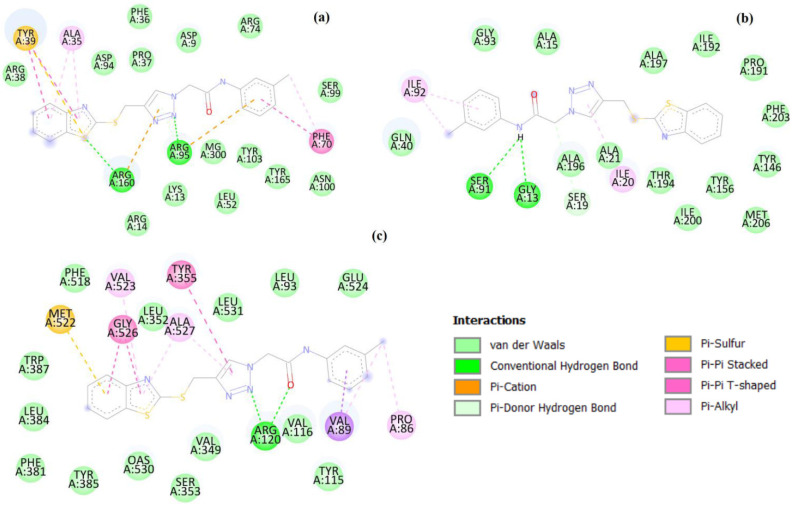
Two-dimensional illustrations of the interaction types between **5d** and protein targets, 1G3U (**a**), 1QSG (**b**), and 5F19 (**c**).

All compounds **5a**–**f** can bind to the active site of the protein targets. The synthesized compounds can form hydrogen bonds (H-bond) with the amino acids in the active site of the protein targets. The number of H-bonds of **5a**–**f** with the related protein targets is collected in Table 6. H-bond interactions were found between nitrogen atoms on the triazole ring of the compounds **5a**–**f** and amino acids in the active site of the protein targets, indicating that the triazole unit of the synthesized compounds could be the key to binding and inhibiting the protein (Figure 6).

**Figure 7:**
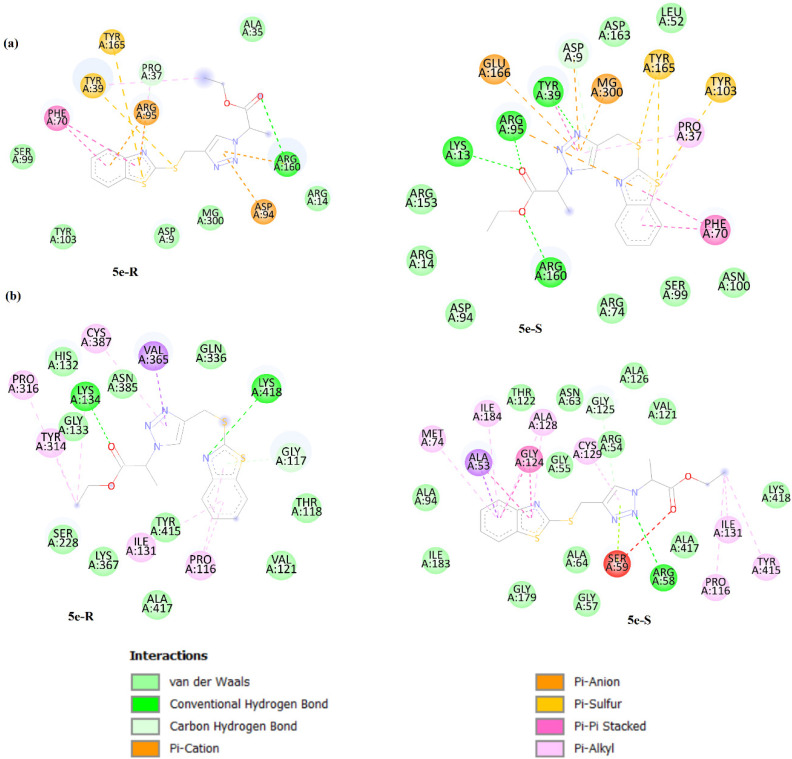
Two-dimensional illustrations of the interaction types between **5e-R** and **5e-S** and protein targets, 1G3U (**a**) and 4P8N (**b**).

It is interesting to note that the compound **5e** has two enantiomers (**5e-R** and **5e-S**). Molecular docking studies of these enantiomers can be interesting. Except for the protein 1QSG, the binding energy of **5e-S** with other proteins is higher than **5e-R** (Table 6). For example, we considered only the interaction of **5e-R** and **5e-S** with 1G3U and 4P8N. For 1G3U, the **5e-R** interacts with ARG-160 through a hydrogen bond, one carbon–hydrogen interaction with PRO-37, six van der Waals interactions with SER-99, TYR-103, ASP-9, MG-300, ARG-14, and ALA-35, and several other interactions such as P–cation, Pi–anion, Pi–sulfur, Pi–Pi stacked, and Pi–alkyl. **5e-S** shows more interactions, specifically four hydrogen bonds with LYS-13, ARG-95, TYR-39, and ARG-160. Moreover, it also has a carbon–hydrogen bond with ASP-9. The **5e-S** molecule also has eight van der Waals interactions with ARG-153, ARG-14, ASP-94, ARG-74, SER-99, ASN-100, LEU-52, and ASP-162. Like **5e-R**, **5e-S** also interacts with the active site, interactions such as Pi–cation, P–anion, Pi–sulfur, Pi–Pi stacked, Pi–Pi T-shaped, and Pi–alkyl. These results could indicate the higher binding affinity of **5e-S** in comparison to **5e-R** (Figure 7). Based on the interaction results for these enantiomers for 4P8N, **5e-R** has two hydrogen bonds with LYS-134 and LYS-418 and six alkyl and Pi–alkyl interactions with PRO-316, CYS-387, TYR314, ILE-131, and PRO-116. There are also a Pi–donor hydrogen bond with GLY-117 and nine van der Waals interaction with TYR-415, HIS-132, GLY-133, GLN-336, THR-118, VAL-121, ALA-417, LYS-367, and SER-228. Moreover, the **5e-R** molecule has a Pi–sigma interaction with VAL-365. Molecule **5e-S** has one hydrogen bond with ARG-58 and eight alkyl and Pi–alkyl interactions with MET-74, ILE-184, ALA-128, ILE-131, PRO-116, CYX-129, and TYR-415. Furthermore, there are one carbon–hydrogen bond with GLY-125 and thirteen van der Waals interaction with LYS-418, ILE-183, ALA-94, THR-122, GLY-55, ASN-63, ARG-54, ALA-126, VAL-121, ALA-417, GLY-57, ALA-64, and GLY-179. These are some other interactions that **5e-S** creates with the protein (Figure 7).

The Lipinski, Pfizer, and GSK rules are the focus of the ADMET study. While the molecules **5a**, **5b**, and **5d** showed the best docking results, the ADMET analysis reveals that all three mentioned molecules passed two out of the three rules above. The control molecule for 4ZE2, Posaconazole, passes only the Pfizer rule, suggesting that the molecules **5a**, **5b**, and **5d** have a better chance than others, mainly molecule **5d**, owing to its robust docking against anti-inflammatory and anti-tuberculosis targets. The ADMET results are summarized in Table 6.

Lipophilicity (permeability of a drug to reach the target tissue in the body) and solubility are two important molecular properties for the absorption of a drug. The calculated solubility in water (Log S) of the synthesized compounds **5a**–**f** followed the order: **5f** > **5e** > **5a** > **5b** > **5d** > **5c**. The estimated lipophilicity (Log P) also followed the order: **5c** > **5d** > **5b** > **5a** > **5e** > **5f**. After analyzing the chemical structures of **5a**–**f** (Scheme 2), it is easy to understand that, as the number of hydrocarbon chains increases, the solubility in water decreases and lipophilicity increases.

## 3. Experimental Section

### 3.1. General Information

The chemicals and solvents were purchased from Sigma-Aldrich (St. Louis, MO. USA), Fluka (Neu-Ulm, Germany), and Merck (Darmstadt, Germany). The structure of the synthesized triazole derivatives **5a**–**f** was confirmed by ^1^H NMR, ^13^C NMR, and CHN elemental data. A Bruker (DRX-500 Avance) NMR was used to record the ^1^H and ^13^C NMR spectra in CDCl_3_ and DMSO at room temperature (The Supplementary Material). The multiplicity of the proton signals is abbreviated with s for singlet, d for doublet, m for multiplet, and dd for the doublet of doublet peaks. Quantitative microanalyses (CHN) of the products **5a**–**f** were carried out on a Thermo Finnigan Flash-1112EA microanalyzer. 

### 3.2. Computational Details

Geometric optimization of the characterized structure **5e** was performed at the DFT/B3LYP level using the 6-31 + G(d, *p*) basis set utilizing the Gaussian 09 software [64]. The vibrational frequencies were calculated simultaneously to ensure that each stationary point was a real minimum. NMR data calculations were performed using the gauge-independent atomic orbital (GIAO) method [65]. Relative chemical shifts were calculated using the corresponding tetramethylsilane for ^1^H NMR and ^13^C NMR shielding, calculated at the same level of theory as the reference. Further, the solvent effects were taken into account by using the conductor-like polarizable continuum model (CPCM) [66] through DFT calculations performed at the B3LYP/6-31 + G(d, *p*) level of theory in DMSO as the solvent.

### 3.3. Molecular Docking and ADMET Details

Molecular docking is a crucial component of the in silico study of drug discovery and development. We performed in silico docking studies with Pyrx to demonstrate the synthesized compounds’ anti-tuberculosis, anti-fungal, and anti-inflammatory properties. Pyrx was used to determine the interactions between the studied compounds and the specific protein targets [67]. All the molecules’ smiles were extracted by Cheminfo [68]. Afterward, all the structures were minimized using chimera and saved as PDB files. The next step involved unifying the six PDB structures using OpenBabel and converting them to a single SDF file [69]. RSCB PDB was used to extract all protein structures. Chimera was applied in all protein structures to remove solvents, water molecules, unnecessary ions, and ligands [70]. We selected a single control molecule per protein target for molecular docking to compare our synthesized molecules **5a–f** with the reference molecules. All the procedures to prepare the reference molecules for docking were the same as for **5a**–**f**, as shown above. Each target protein’s PDB ID and the control molecules are listed below in Table 7. Pyrx was then used to dock the preprocessed data. For the ADMET approach, we used the ADMETlab2 web server to analyze and predict the synthesized molecules’ properties [71]. In this section, all synthesized molecules **5a–f** and control molecules are converted into their smile strings and fed to ADMETlab2.

### 3.4. General Synthesis Procedure for 2-Mercaptobenzothiazole ***2***

To a solution of 2-aminothiophenol **1** (1 mmol) in 30 mL of KOH solution (10%), CS_2_ (6 mmol) was added and refluxed for three hours. The mixture was cooled, then 25 mL water and 25 mL ethanol were added and heated until 40 °C. At this temperature, 4.5 mL acetic acid was added dropwise to the reaction mixture to adjust the pH = 6. The mixture was cooled to room temperature, filtrated, and dried overnight to obtain the desired 2-mercaptobenzothiazole **2** in 90% yield.

### 3.5. General Synthesis Procedure for S-Propargylated Mercaptobenzothiazole ***3***

To a solution of 2-mercaptobenzothiazole **2** (5 mmol) in 10 mL dioxane, Et_3_N (5 mmol) was added and stirred at room temperature for 45 min. Propargyl bromide (5 mmol, 80% in toluene) was added dropwise to the reaction mixture and refluxed at 45 °C for one hour. After completion of the reaction, the mixture was cooled, and then, 20 mL of water was added. The reaction mixture was extracted by DCM (3 × 5 mL). The organic layer was dried over Na_2_SO_4_, filtrated, and evaporated to give the desired S-propargyl mercaptobenzothiazole **3** in 95% yield.

### 3.6. General Synthesis Procedure for 1,2,3-Triazole Derivatives ***5a***–***f***

To a solution of *t*-BuOH/H_2_O (1:1, 5 mL), α-halo ester/amide **4** (1 mmol) and NaN_3_ (1.3 mmol) were added and stirred at room temperature for one hour, then CuSO_4_ and sodium ascorbate (50% mol) and S-propargyl mercaptobenzothiazole 3 (1 mmol) were added. The reaction mixture was stirred at room temperature for 12 h. After completion of the reaction, as monitored by TLC, 10 mL of water was added, and the mixture was extracted with DCM (3 × 5 mL). The organic layer was dried over Na_2_SO_4_, filtrated, and evaporated under a vacuum. The desired products **5a**–**f** were purified by column chromatography using ethyl acetate/hexane (1:1). The structure of the synthesized triazole derivatives **5a**–**f** was confirmed by ^1^H and ^13^C NMR spectra data and CHN analysis.

## 4. Conclusions

We successfully reported synthesizing a series of new 1,2,3-triazoles based on benzothiazole by the 1,3-dipolar cycloaddition reaction of *S*-propargyl mercaptobenzothiazole and α-halo ester/amide in moderate to good yields. This protocol is easy and green and proceeds under mild reaction conditions with available starting materials. We also studied the full structural, electronic, and NMR data of the synthesized compound **5e** using the B3LYP/6-31 + G(d, *p*) computational method. Molecular docking and ADMET studies indicate that the **5d** scaffold could be the key to binding with the protein targets more than the other five synthesized molecules in terms of their anti-inflammatory and anti-tuberculosis activity. All synthesized molecules did not achieve a better docking result in terms of antifungal activity than the reference molecules; however, **5a** has the same binding affinity for 4ZE2 as the control molecule, suggesting that this molecule may possess antifungal properties. Further in vitro analysis is needed to prove the in silico results.

## Figures and Tables

**Table 1:** Optimization of catalyst, solvent, and temperature for the synthesis of **5a** ^a^.

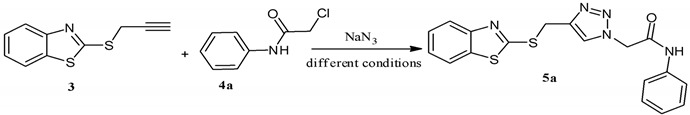				
Entry	Catalyst	Solvent	T (°C)	Yield
1	CuCl (50 mol%)	H_2_O	60	trace
2	CuI	H_2_O/EtOH	60	14
3	CuSO_4_·5H_2_O (50 mol%)/sodium ascorbate (50 mol%)	H_2_O	60	trace
4	CuSO_4_·5H_2_O (50 mol%)/sodium ascorbate (50 mol%)	H_2_O/*t*-BuOH	r.t	75
5	CuSO_4_·5H_2_O (50 mol%)/sodium ascorbate (50 mol%)	H_2_O/*t*-BuOH	60	30
6	CuCl (50 mol%)	H_2_O/*t*-BuOH	r.t	22

^a^ Reaction conditions: solvent (5 mL, 1:1), **3** (1 mmol), **4a** (1 mmol), NaN_3_ (1.3 mmol), catalyst (50 mol%), reaction time 12 h.

**Table 3:** The calculated structural parameters of **5e** by the B3LYP/6-31 + G(d, *p*) method.

Geometrical Parameters ^a^	Theoretical Value
Bond lengths (Å)	
C^1^—C^2^	1.397
C^2^—S^12^	1.757
N^13^—C^11^	1.298
C^11^—S^14^	1.761
S^14^—C^15^	1.856
N^21^—N^22^	1.306
C^30^—O^39^	1.215
C^38^—C^31^	1.463
C^20^—C^18^	1.382
Bond angles (°)	
C^1^—C^2^—C^3^	121.7
H^10^—C^6^—C^5^	119.7
C^11^—S^14^—C^15^	102.8
N^21^—N^22^—N^23^	107.6
N^23^—C^24^—H^25^	105.2
H^16^—C^15^—H^17^	109.8
Dihedral angles (°)	
C^11^—S^14^—C^15^—H^17^	58.5
H^37^—C^34^—C^31^—H^32^	61.1
O^39^—C^30^—C^24^—H^29^	85.5

^a^ For the numbering of atoms, refer to Figure 1.

**Table 4:** The electronic data at the B3LYP/6-31 + G(d, *p*) level for **5e** in DMSO.

Parameter	Value
Energy (Hartree)	−1747.11
μ_D_ (Deby)	8.26
E_HOMO_ (eV)	−6.32
E_LUMO_ (eV)	−1.34
E_g_ (eV)	4.98
Chemical potential (μ) (eV)	−3.83
Chemical hardness (η) (eV)	2.49
Electrophilicity (ω) (eV)	2.94

**Table 5:** Binding energy data for the triazoles **5a**–**f** and control molecules (in kcal mol^−1^).

Compound ID	Protein PDB ID					
	1G3U	1QSG	4P8N	5F19	5V3X	4ZE2
**5a**	−9.6	−8.5	−9.1	−9.3	−9.4	−9.4 (1) ^a^
**5b**	−8.2	−9.0	−9	−9.2	−9.2	−9.0
**5c**	−9.3	−8.8	−9.1	−9.6	−8.5	−8.9
**5d**	−9.7 (2)	−9.1	−9.5 (1)	−9.7 (1)	−9.7 (3)	−9.4
**5e-R**	−7.3	−7.8	−7.7	−7.6	−7.8	−7.6
**5e-S**	−9.3	−7.7	−9.2	−8.3	−7.8	−8.5
**5f**	−6.3	−8	−7.7	−8	−6.8	−7.7
Isoniazid ^b^	−6.5	−5.8	-	-	-	-
BTZ043 ^b^	-	-	−10.6	-	-	-
Ibuprofen ^b^	-	-	-	−7.4	-	-
I28 ^b^	-	-	-	-	−10.1	
Posaconazole ^b^	-	-	-	-	-	−10.1

^a^ Number of H-bonds; ^b^ control molecules.

**Table 6:** A summary of the ADMET properties for **5a**–**f** and the control molecules.

Compound	Log S ^a^	Log P ^b^	Pfizer Rule	Lipinski Rule	GSK Rule
**5a**	−4.305	3.455	Rejected	Accepted	Accepted
**5b**	−4.463	3.518	Accepted	Accepted	Rejected
**5c**	−4.892	4.127	Rejected	Accepted	Rejected
**5d**	−4.607	3.912	Rejected	Accepted	Accepted
**5e**	−3.944	3.127	Rejected	Accepted	Accepted
**5f**	−3.362	2.257	Accepted	Accepted	Accepted
Isoniazid	−0.495	−0.813	Accepted	Accepted	Rejected
BTZ043	−5.876	3.713	Accepted	Rejected	Accepted
Ibuprofen	−3.701	3.687	Rejected	Accepted	Accepted
I28	−4.885	5.264	Rejected	Rejected	Accepted
Posaconazole	−3.014	1.451	Accepted	Rejected	Rejected

^a^ Solubility in water; ^b^ lipophilicity.

**Table 7:** Protein names, the related PDB ID, and their control molecules for docking study.

Protein Name	PDB ID	Control Molecule
DPRE1	4P8N	BTZ043
Saccharomyces cerevisiae CYP51	4ZE2	Posaconazole
COX-2	5F19	Ibuprofen
Pks13	5V3X	I28
Anti-tuberculosis target protein ENR	1QSG	Isoniazid
Thymidylate kinase of Mycobacterium tuberculosis	1G3U	Isoniazid

## Data Availability

Not applicable.

## Supplementary Materials

The following supporting information can be downloaded at: https://www.mdpi.com/article/10.3390/molecules27238555/s1, Spectral data of the 1,2,3-triazoles **5a**–**f**.

## Sample Availability

Samples of the compounds are not available from the authors.
