# Supplementary material for: Novel 1,2,3-Triazole-Based Benzothiazole Derivatives: Efficient Synthesis, DFT, Molecular Docking, and ADMET Studies
Source: Molecules. 2022 Dec 5;27(23):8555. doi: 10.3390/molecules27238555 (PMC9740823; doi:10.3390/molecules27238555)
Supplement: Supplementary file 1 [file molecules-27-08555-s001.zip › molecules-2045161-supplementary.pdf]

Novel 1,2,3-Triazole-Based Benzothiazole Derivatives: Efficient Synthesis, DFT, Molecular Docking, and ADMET Studies

Zohreh Mirjafary [*a], Mahdieh Mohammad Karbasi [a], Parsa Hesamzadeh[a], Asghar Amiri[b], Hamid Reza Shaker [a], Hamid Saeidian [b]

[a] *Department of Chemistry, Science and Research Branch, Islamic Azad University, Tehran, Iran*

[b] *Department of Science, Payame Noor University (PNU), PO Box: 19395-4697, Tehran, Iran*

Spectral data of the 1,2,3-triazoles 5a-f

2-(4-((benzo[d]thiazol-2-ylthio)methyl)-1H-1,2,3-triazol-1-yl)-N-phenylacetamide (**5a**): ^{1}H NMR (500 MHz, DMSO): δ = 4.75 (s, 2H), 5.32 (s, 2H), 7.07 (t, *J* = 7.4 Hz, 1H), 7.32 (t, *J* = 7.7 Hz, 2H), 7.37 (t, *J* = 7.7 Hz, 1H), 7.47 (t, *J* = 7.7 Hz, 1H), 7.58 (d, *J* = 8.5 Hz, 2H), 7.92 (d, *J* = 8.0 Hz, 1H), 8.02 (d, *J* = 8.0 Hz, 1H), 8.15 (s, 1H), 10.46 (s, 1H) ppm. ^{13}C NMR (125 MHz, DMSO): δ = 27.41, 52.24, 119.20, 121.26, 121.79, 123.74, 124.51, 125.54, 126.34, 128.87, 134.74, 138.37, 142.15, 152.60, 164.10, 165.83 ppm. Anal. Calcd for $C_{18}H_{15}N_5OS_2$: C, 56.67; H, 3.96; N, 18.36. found: C, 56.73; H, 4.01; N, 18.46.

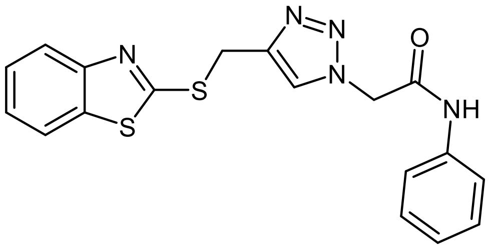

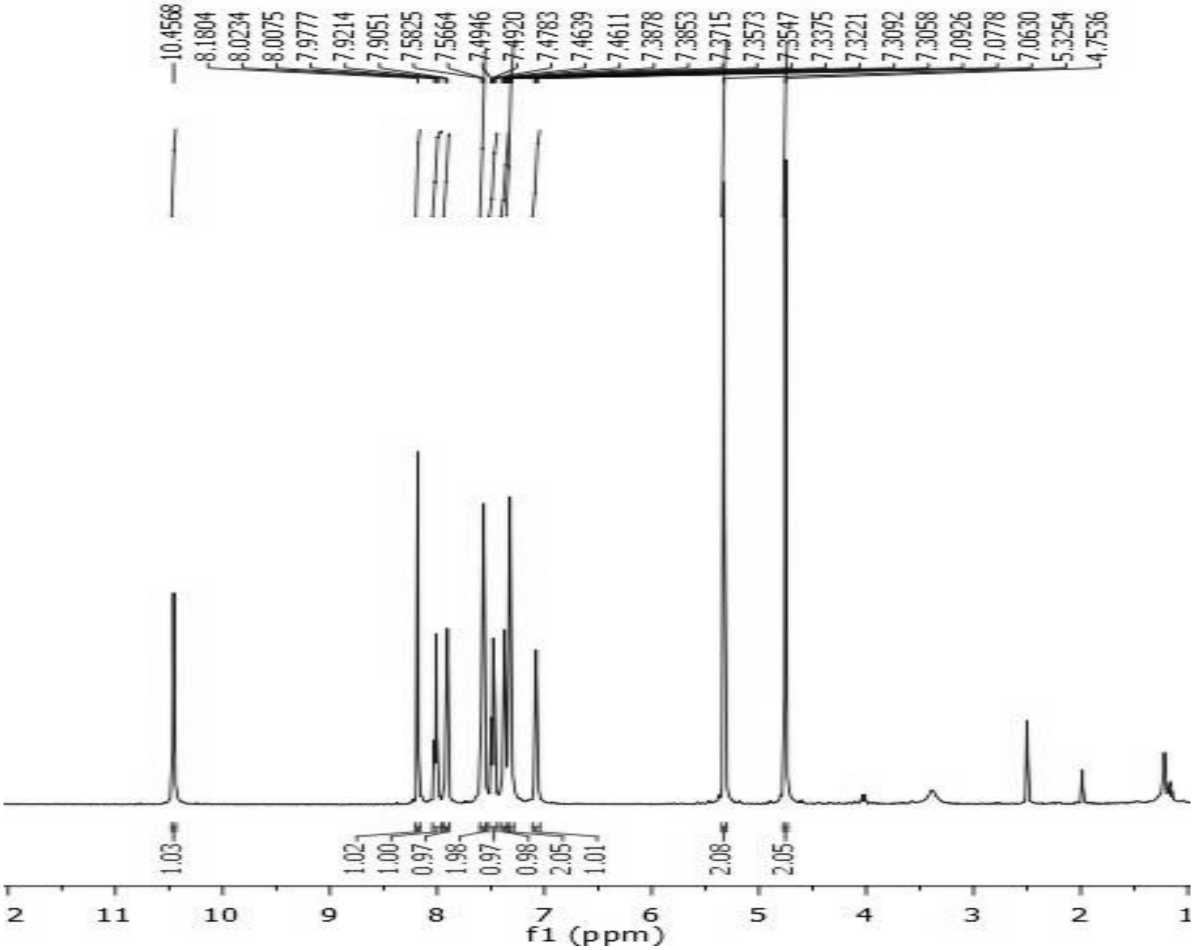

10.4568
8.1804
8.0234
8.0075
7.9777
7.9214
7.9051
7.5825
7.5664
7.4946
7.4920
7.4783
7.4639
7.4611
7.3878
7.3853
7.3715
7.3573
7.3547
7.3375
7.3221
7.3092
7.3058
7.0926
7.0778
7.0630
5.3254
4.7536
1.03
1.02
1.00
0.97
1.98
0.97
0.98
2.05
1.01
2.08
2.05
f1 (ppm)

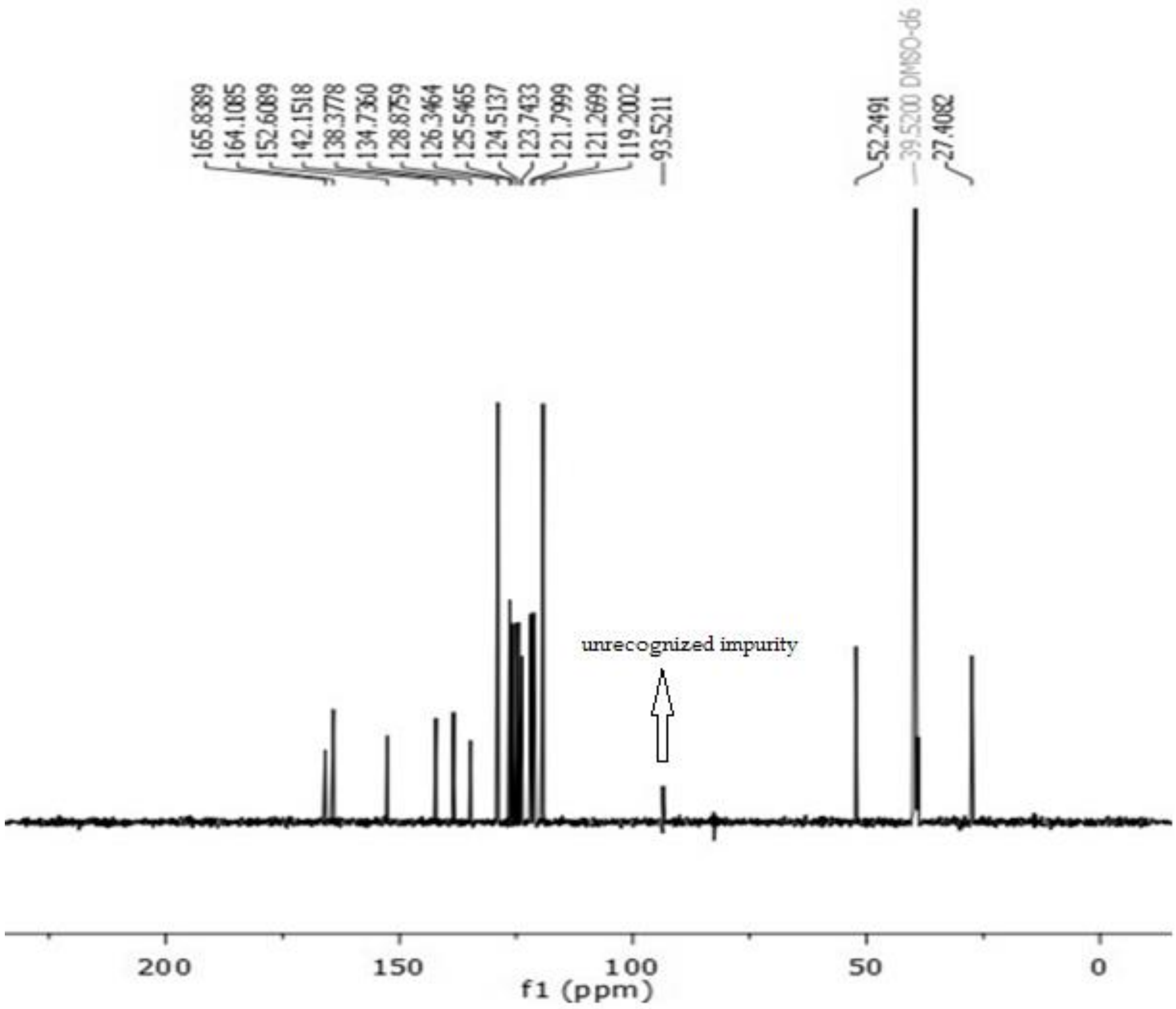

2-(4-((benzo[d]thiazol-2-ylthio)methyl)-1H-1,2,3-triazol-1-yl)-N-phenylacetamide (**5b**): ^{1}H NMR (500 MHz, DMSO): δ = 3.72 (s, 3H), 4.75 (s, 2H), 5.27 (s, 2H), 6.91 (d, *J* = 9.1 Hz, 2H), 7.38 (t, *J* = 7.3 Hz, 1H), 7.50-7.46 (m, 3H), 7.92 (d, *J* = 8.0 Hz, 1H), 8.04 (d, *J* = 8.0 Hz, 1H), 8.16 (s, 1H), 10.34 (s, 1H) ppm. ^{13}C NMR (125 MHz, DMSO): δ = 27.85, 52.61, 55.60, 114.44, 121.23, 121.72, 122.26, 124.98, 125.97, 126.82, 131.90, 135.18, 142.58, 153.05, 155.98, 164.02, 166.30 ppm. Anal. Calcd for $C_{19}H_{17}N_5O_2S_2$: C, 55.46; H, 4.16; N, 17.02. found: C, 55.57; H, 4.09; N, 17.13.

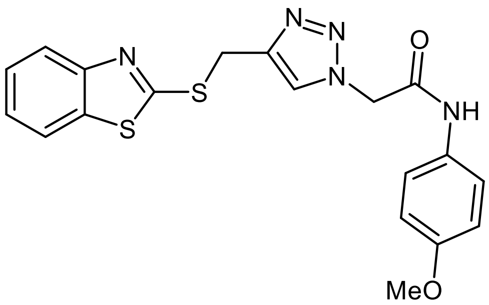
N
S
S
N=N
N
O
NH
MeO

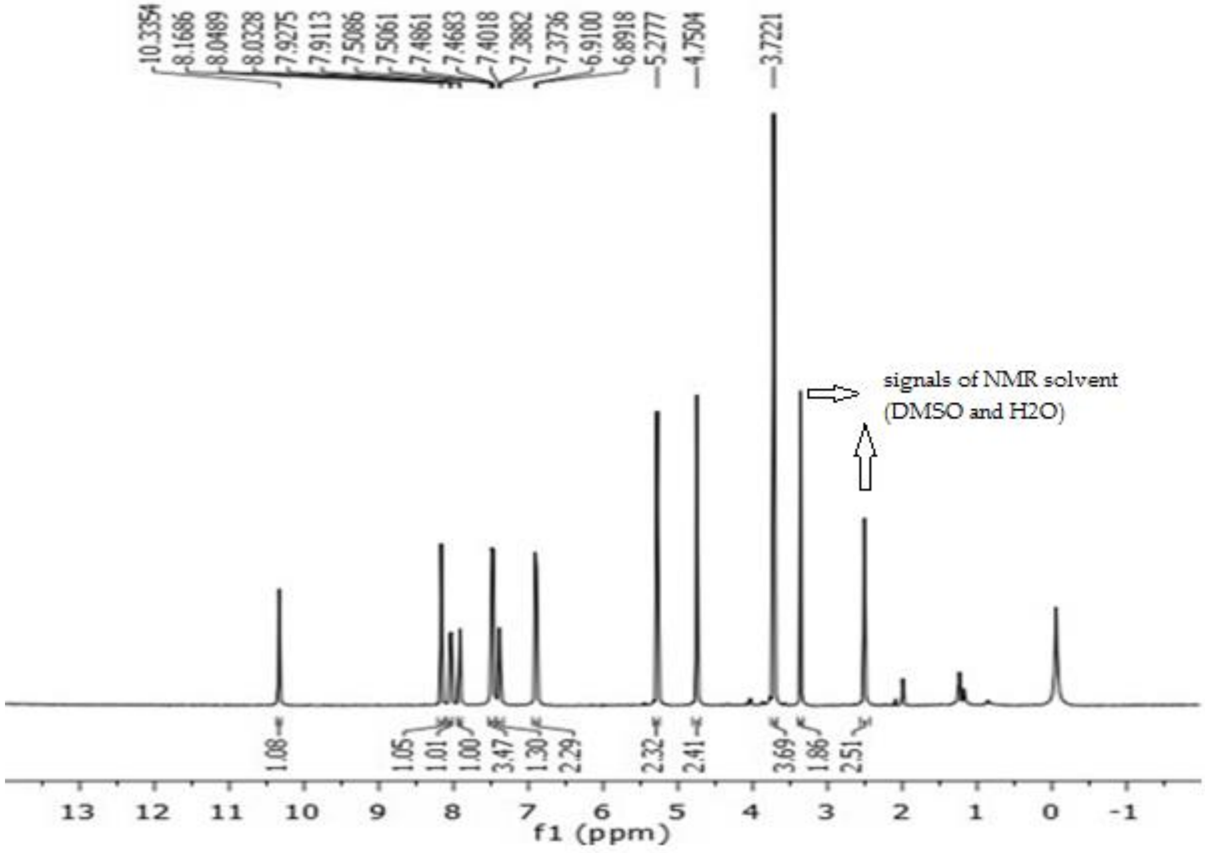
10.3354
8.1686
8.0489
8.0328
7.9275
7.9113
7.5086
7.5061
7.4861
7.4683
7.4018
7.3882
7.3736
6.9100
6.8918
5.2777
4.7504
3.7221
signals of NMR solvent
(DMSO and H2O)
1.08
1.05
1.01
1.00
3.47
1.30
2.29
2.32
2.41
3.69
1.86
2.51
13
12
11
10
9
8
7
6
5
4
3
2
1
0
-1
f1 (ppm)

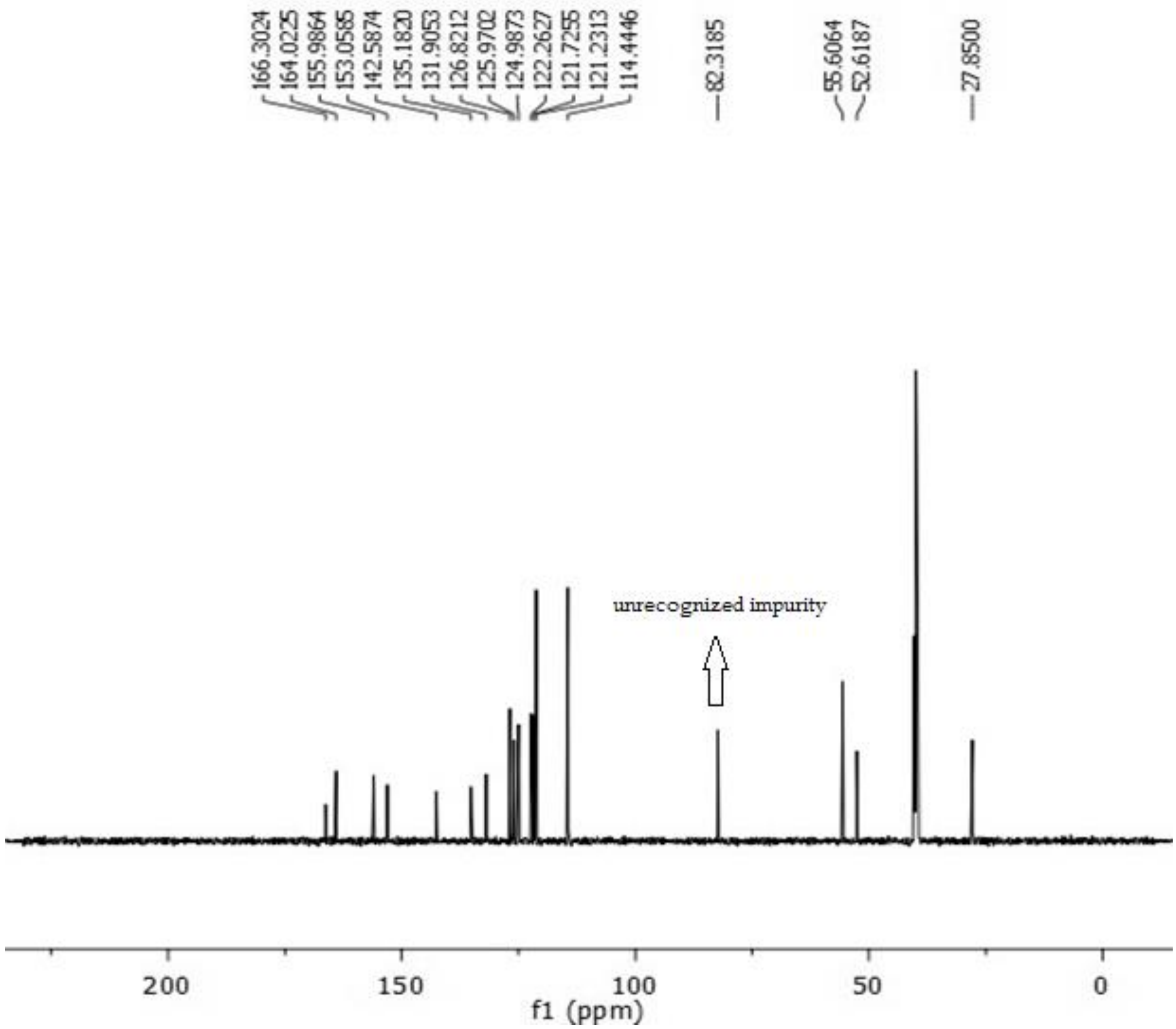

2-(4-((benzo[d]thiazol-2-ylthio) methyl)-1H-1,2,3-triazol-yl)-N-(3-chlorophenyl)acetamide (**5c**): ^{1}H NMR (500 MHz, DMSO): δ = 4.73 (s, 2H), 5.33 (s, 2H), 7.14 (d, *J* = 8.7 Hz, 1H), 7.36 (dd, J_1 = 16.5 Hz, J_2 = 8.4 Hz, 2H), 7.42 (d, *J* = 7.7 Hz, 1H), 7.46 (t, *J* = 7.6 Hz, 1H), 7.74 (s, 1H), 7.90 (d, *J* = 8.2 Hz, 1H), 8.01 (d, *J* = 8.0 Hz, 1H), 8.16 (s, 1H), 10.63 (s, 1H) ppm. ^{13}C NMR (125 MHz, DMSO): δ = 27.39, 52.33, 117.63, 118.74, 121.28, 121.82, 123.51, 124.54, 125,57, 126.37, 130.63, 133.19, 134.74, 139.78, 142.22, 152.81, 164.60, 165.83 ppm. Anal. Calcd for $C_{18}H_{14}ClN_5OS_2$: C, 51.98; H, 3.39; N, 16.84. found: C, 52.05; H, 3.44; N, 16.96.

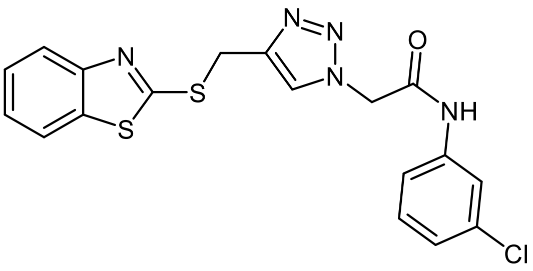

N
N
N
O
S
N
S
NH
Cl

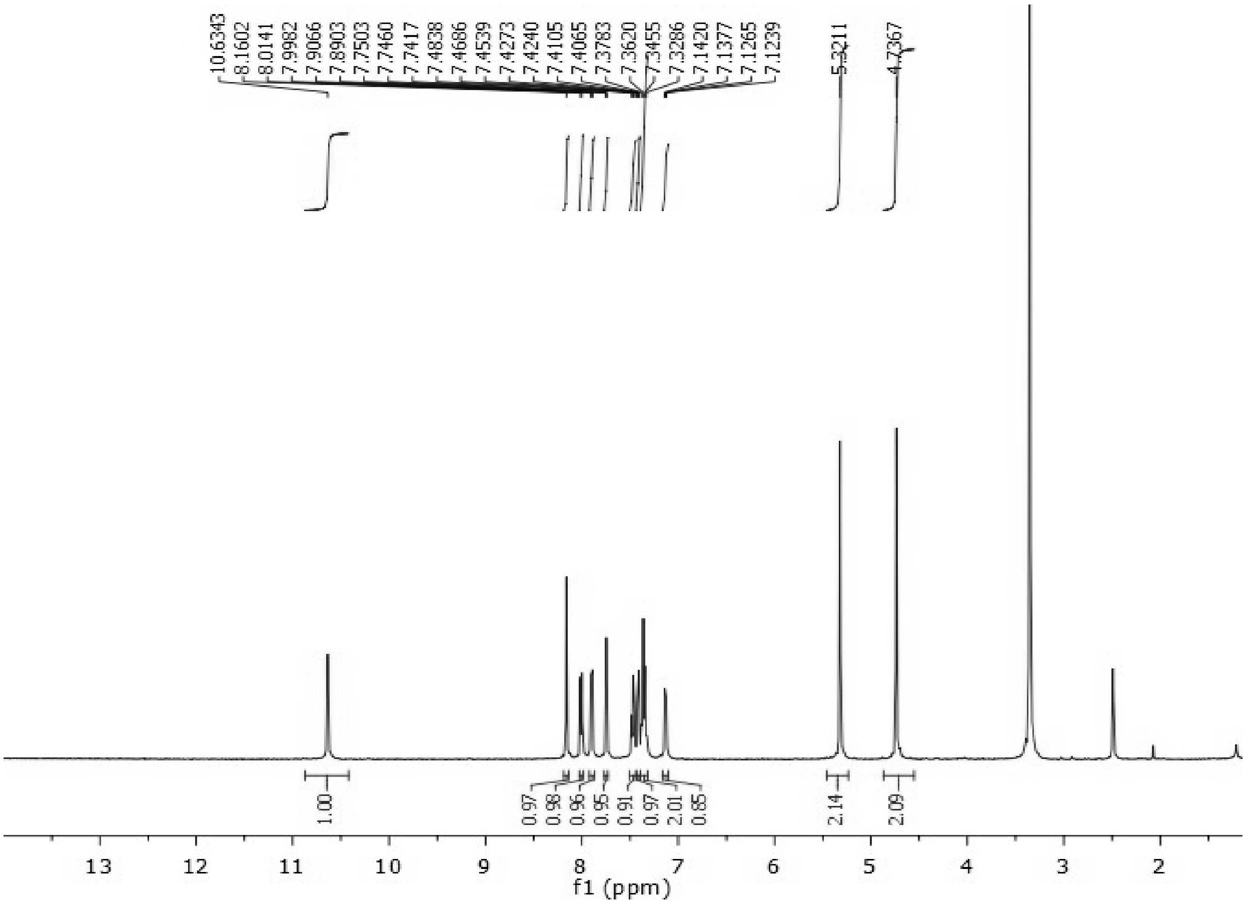

10.6343
8.1602
8.0141
7.9982
7.9066
7.8903
7.7503
7.7460
7.7417
7.4838
7.4686
7.4539
7.4273
7.4240
7.4105
7.4065
7.3783
7.3620
7.3455
7.3286
7.1420
7.1377
7.1265
7.1239
5.3211
4.7367
1.00
0.97
0.98
0.96
0.95
0.91
0.97
2.01
0.85
2.14
2.09
13
12
11
10
9
8
7
6
5
4
3
2
f1 (ppm)

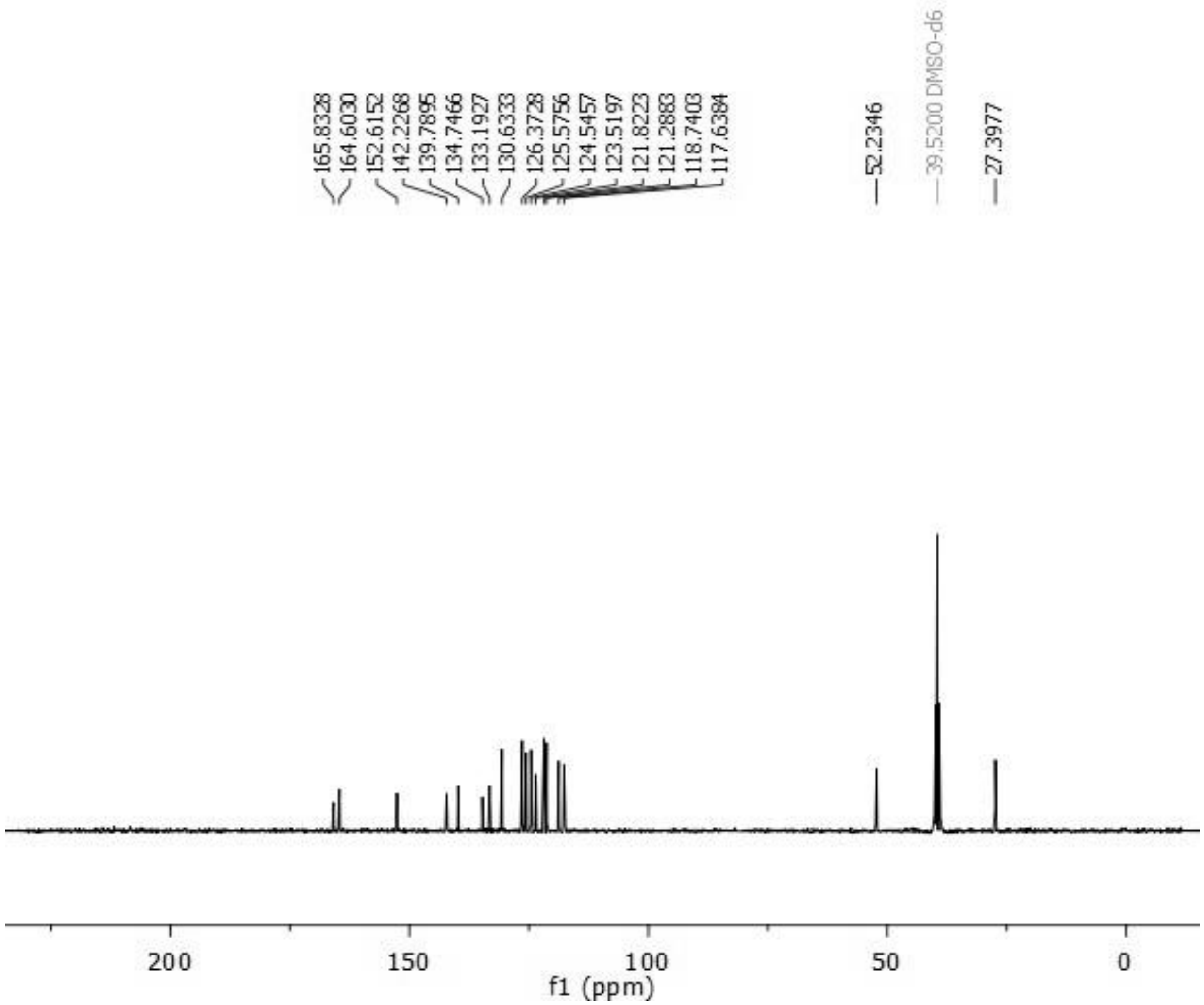

2-(4-((benzo[d]thiazol-2-ylthio) methyl)-1H-1,2,3-triazol-yl)-N-(m-tolyl)acetamide (**5d**): ^{1}H NMR (500 MHz, $CDCl_3$): δ = 2.30 (s, 3H), 4.72 (s, 2H), 5.12 (s, 2H), 6.95 (d, *J* = 7.4 Hz, 1H), 7.17-7.28 (m, 4H), 7.31-7.33 (m, 1H), 7.43 (t, *J* = 7.8 Hz, 1H), 7.74 (d, *J* = 8.1 Hz, 1H), 7.85 (s, 1H), 7.88 (d, *J* = 8.2 Hz, 1H), 9.09 (s, 1H) ppm. ^{13}C NMR (125 MHz, $CDCl_3$): δ = 21.61, 27.66, 53.51, 117.53, 121.08, 121.39, 121.58, 121.77, 124.65, 126.02, 126.35, 128.97, 135.55, 137.00, 139.13, 144.59, 153.02, 163.40, 165.65 ppm. Anal. Calcd for $C_{19}H_{17}N_5OS_2$: C, 57.70; H, 4.33; N, 17.71. found: C, 57.77; H, 4.29; N, 17.68.

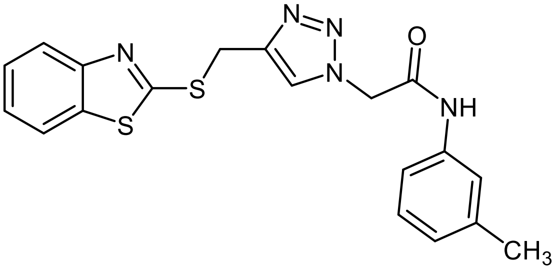

N
S
S
N
N
N
O
NH
CH3

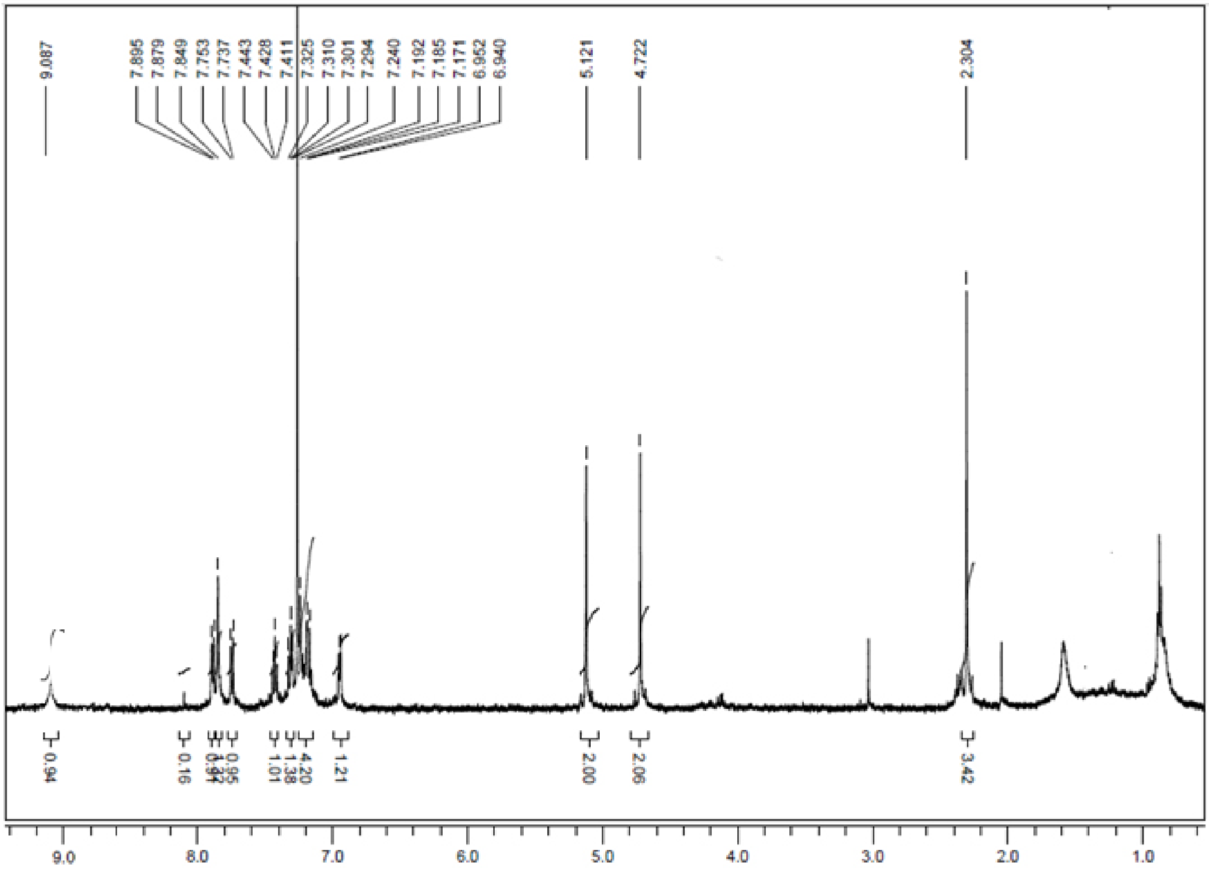

9.087
7.895
7.879
7.849
7.753
7.737
7.443
7.428
7.411
7.325
7.310
7.301
7.294
7.240
7.192
7.185
7.171
6.952
6.940
5.121
4.722
2.304
0.94
0.16
0.95
1.01
1.38
4.20
1.21
2.00
2.06
3.42
9.0
8.0
7.0
6.0
5.0
4.0
3.0
2.0
1.0

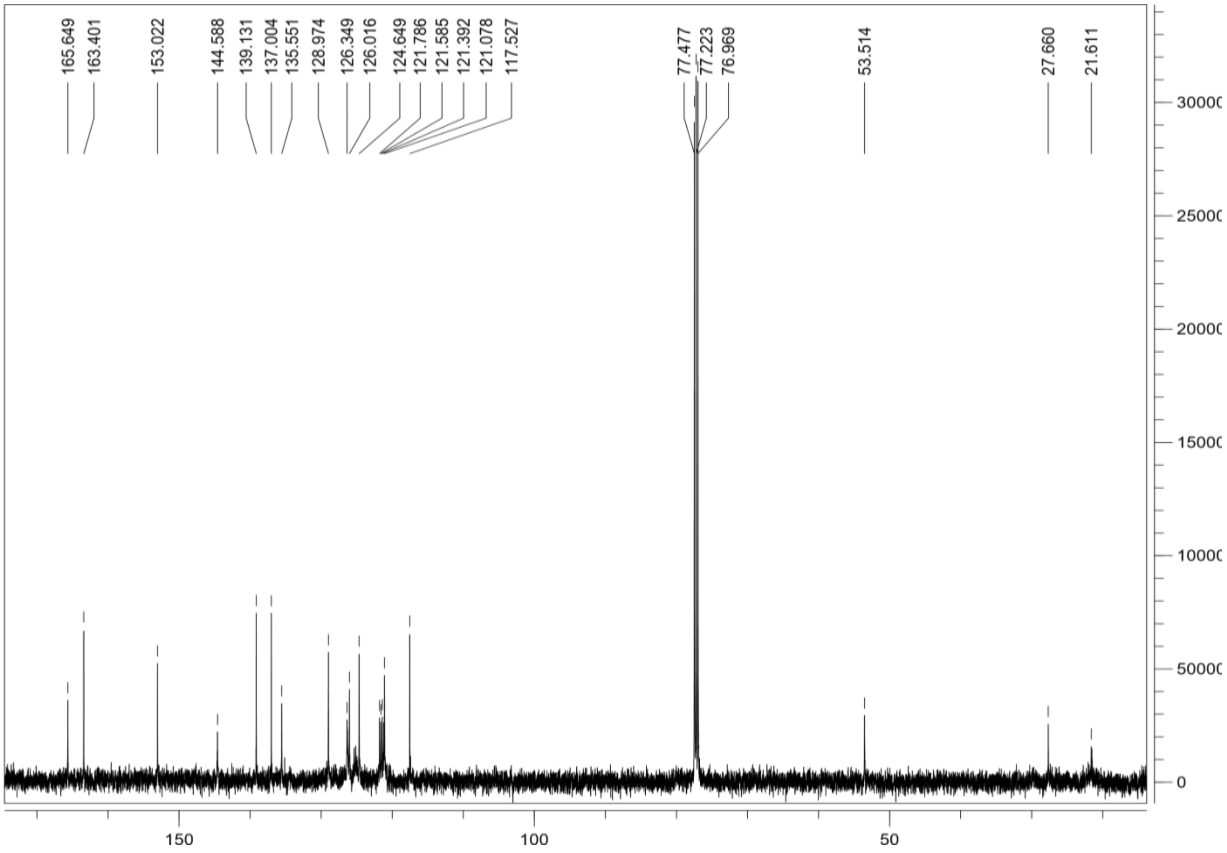

Ethyl 2-(4-((benzo[d]thiazol-2-ylthio)methyl)- 1H-1,2,3-triazol-1-yl)propanoate (**5e**): ^{1}H NMR (500 MHz, DMSO): δ = 1.09 (t, *J* = 7.1 Hz, 3H), 1.72 (d, *J* = 7.3 Hz, 3H), 4.10 (dd, J_1 = 8.6 Hz, J_2 = 5.6 Hz, 2H), 4.71 (s, 2H), 5.61 (dd, J_1 = 8.2 Hz, J_2 = 7.1 Hz, 1H), 7.38 (t, *J* = 7.2 Hz, 1H), 7.47 (t, *J* = 7.2 Hz, 1H), 7.90 (d, *J* = 8.6 Hz, 1H), 8.01 (d, *J* = 8.5 Hz, 1H), 8.24 (s, 1H) ppm. ^{13}C NMR (125 MHz, DMSO): δ = 13.80, 17.05, 27.44, 57.53, 61.66, 121.30, 121.82, 123.85, 124.59, 126.41, 134.76, 142.29, 152.63, 165.74, 169.21 ppm. Anal. Calcd for $C_{15}H_{16}N_4O_2S_2$: C, 51.71; H, 4.63; N, 16.08. found: C, 51.79; H, 4.78; N, 16.14.

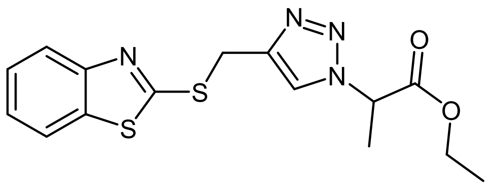
N
S
S
N=N
N
O
O

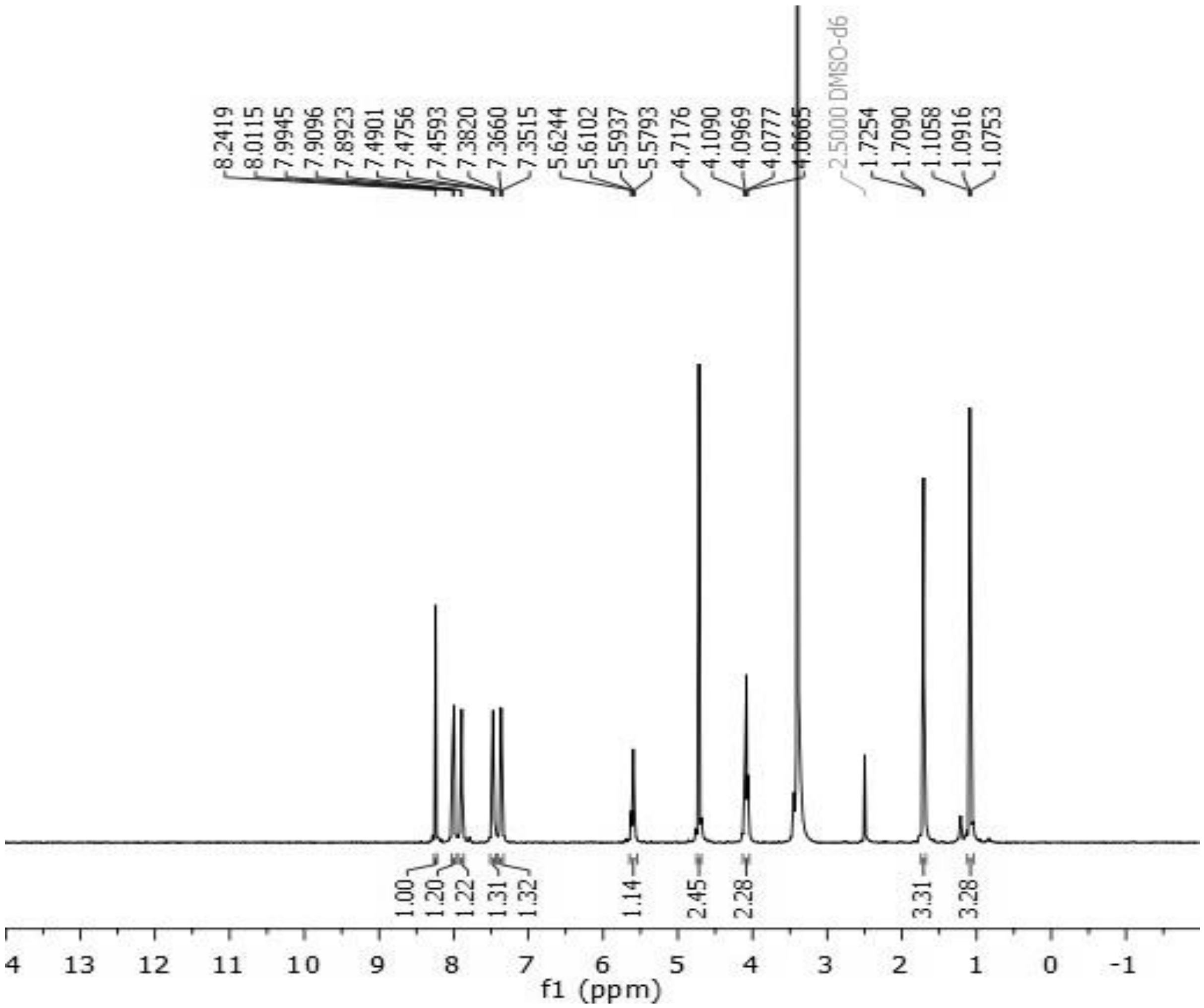
8.2419
8.0115
7.9945
7.9096
7.8923
7.4901
7.4756
7.4593
7.3820
7.3660
7.3515
5.6244
5.6102
5.5937
5.5793
4.7176
4.1090
4.0969
4.0777
4.0665
2.5000 DMSO-d6
1.7254
1.7090
1.1058
1.0916
1.0753
1.00
1.20
1.22
1.31
1.32
1.14
2.45
2.28
3.31
3.28
4
13
12
11
10
9
8
7
6
5
4
3
2
1
0
-1
f1 (ppm)

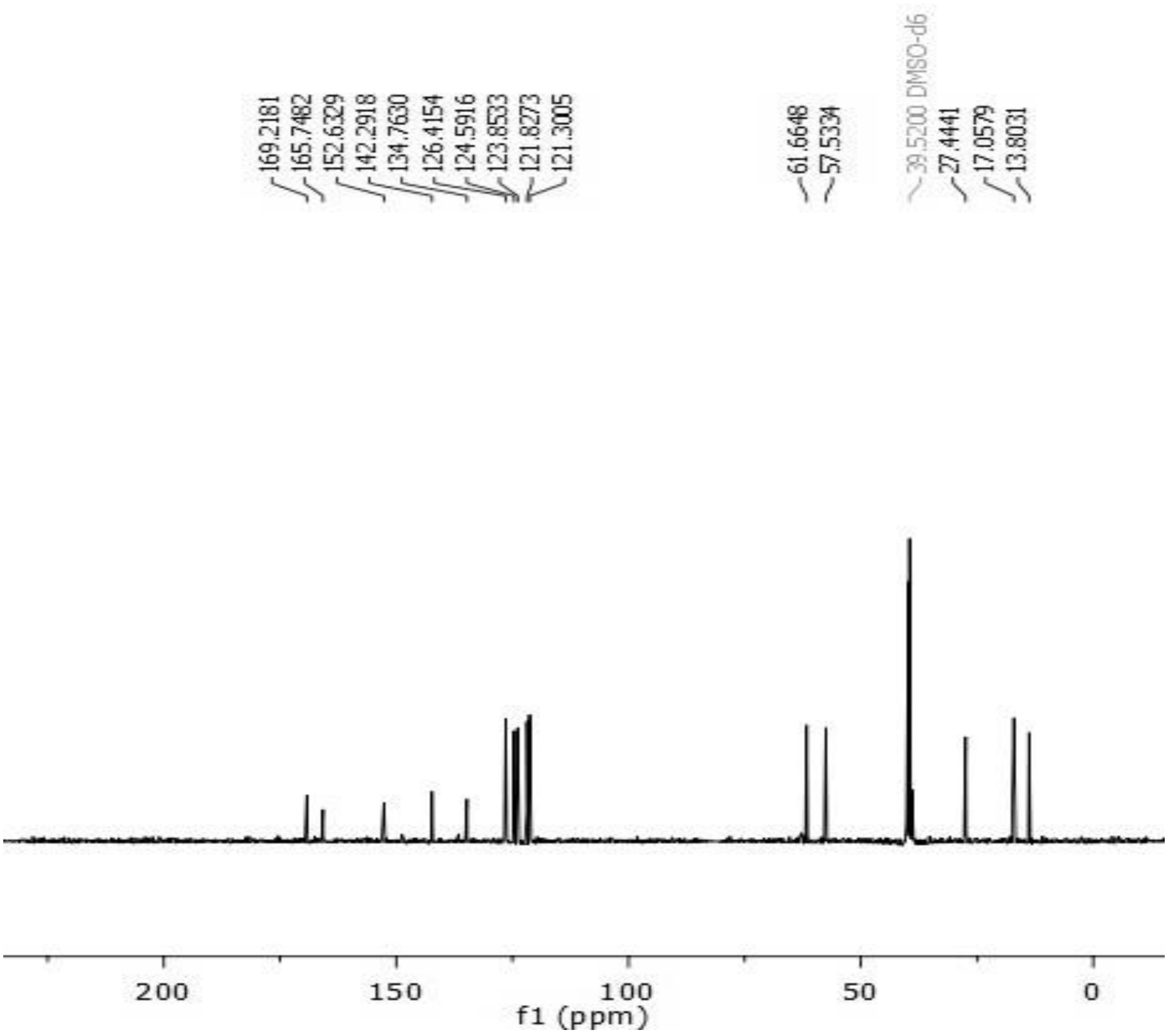

Methyl 2-(4-((benzo[d]thiazol-2-ylthio) methyl)-1H-1,2,3-triazol-1-yl)acetate (**5f**) ^{1}H NMR (500 MHz, DMSO): δ = 3.64 (s, 3H), 4.74 (s, 2H), 5.39 (s, 2H), 7.37 (dt, J_1 = 7.7 Hz, J_2 = 2.9 Hz, 1H), 7.48 (dt, J_1 = 7.8 Hz, J_2 = 1.3 Hz, 1H), 7.89 (dd, J_1 = 8.2 Hz, J_2 = 1.3 Hz, 1H), 8.01 (dd, J_1 = 8.0 Hz, J_2 = 1.3 Hz, 1H), 8.14 (s, 1H) ppm. ^{13}C NMR (125 MHz, DMSO): δ = 27.79, 50.78, 52.95, 121.75, 122.78, 125.00, 125.77, 126.82, 135.21, 142.99, 153.06, 166.21, 168.10 ppm. Anal. Calcd for $C_{13}H_{12}N_4O_2S_2$: C, 48.74; H, 3.78; N, 17.49. found: C, 48.68; H, 3.86; N, 17.56.

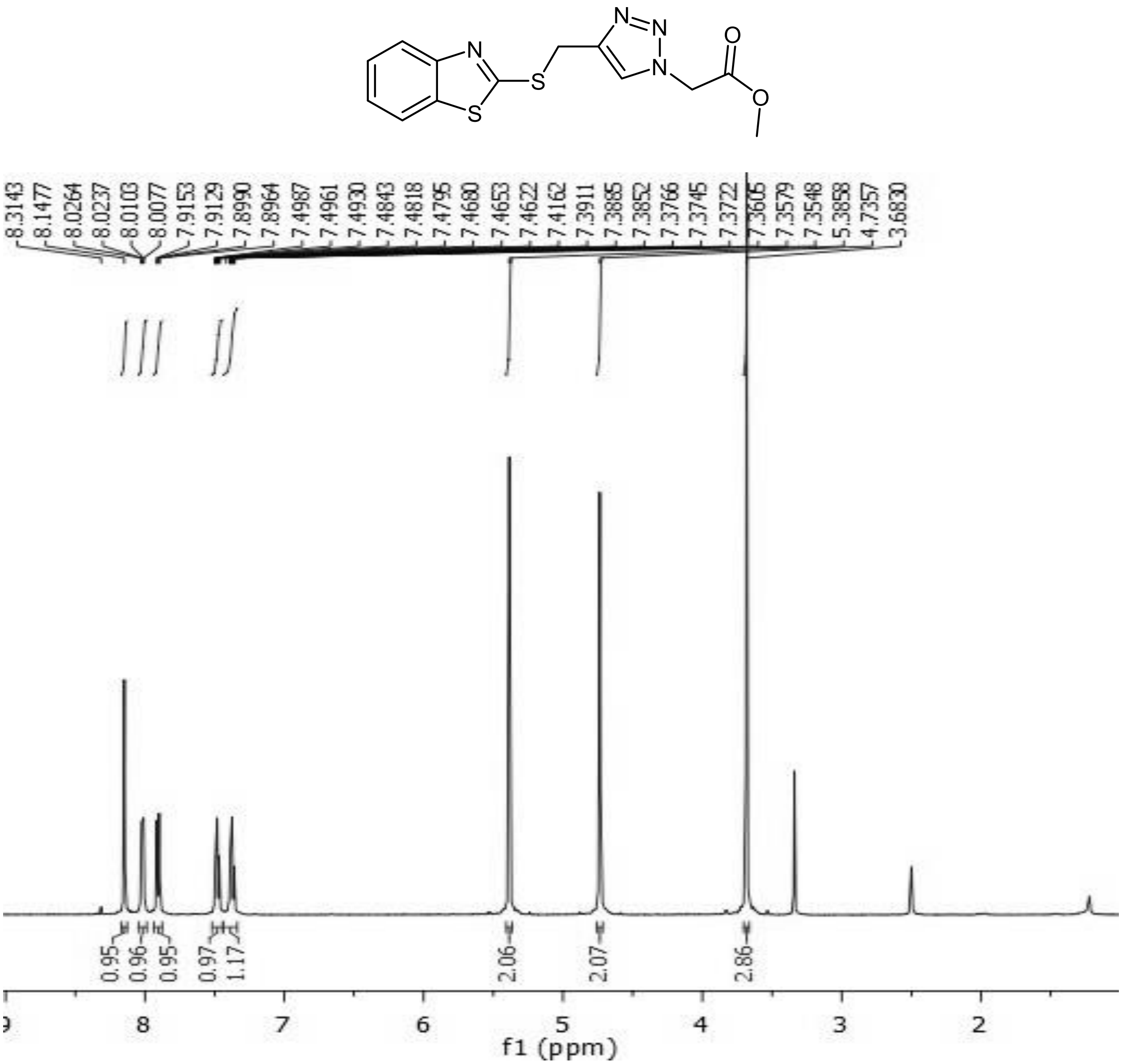

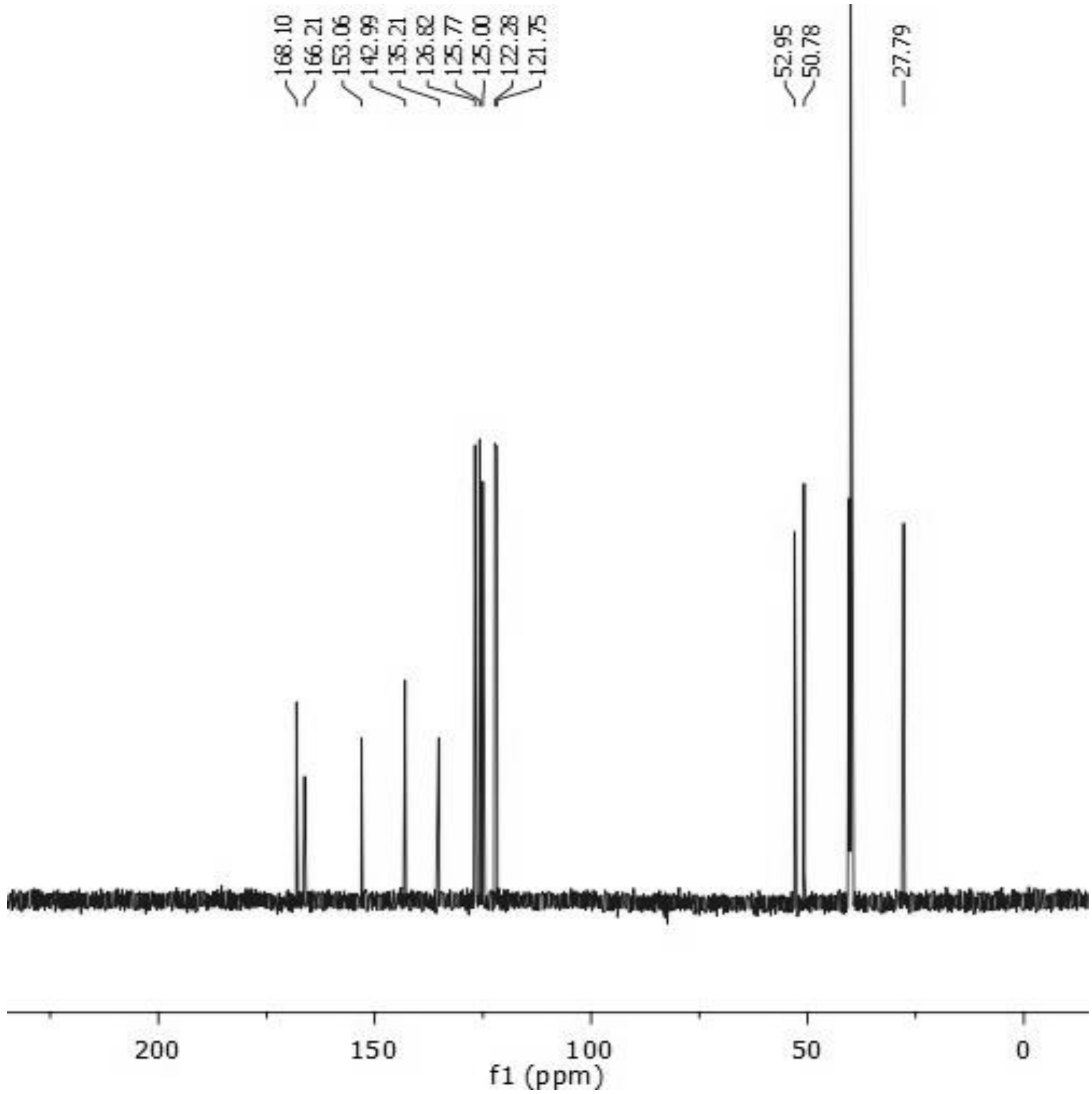
168.10
166.21
153.06
142.99
135.21
126.82
125.77
125.00
122.28
121.75
52.95
50.78
27.79
200
150
100
50
0
f1 (ppm)
